# Annual variation and clinical characteristics of postoperative lower extremity venous thrombosis after urological surgery: a single-center real-world study

**DOI:** 10.3389/fmed.2026.1916959

**Published:** 2026-10-13

**Authors:** Xiaomei Yang, Yang Yin, Yanfei Ma, Yingxin Wang, Haili Long

**Affiliations:** Mianyang Central Hospital, School of Medicine, University of Electronic Science and Technology of China, Mianyang, Sichuan, China

**Keywords:** benign prostatic hyperplasia, lower extremity venous thrombosis, postoperative complication, real-world study, urological surgery

## Abstract

**Objective:**

To analyze the annual variation and clinical characteristics of clinically detected postoperative lower extremity venous thrombosis (LEVT) during hospitalization in patients undergoing urological surgery at our institution from 2022 to 2025, providing reference for perioperative thrombosis risk assessment and preventive management.

**Methods:**

A single-center retrospective real-world study was conducted using the hospital electronic medical record system. Patients who developed LEVT for the first time during hospitalization following urological surgery between January 2022 and December 2025 were included. Lower-extremity ultrasonography was performed based on clinical indications rather than routine systematic screening. Detailed information was extracted for patients with thrombosis, including demographic characteristics, admission-related scores and laboratory tests, surgical details, and thrombosis-related outcomes. Descriptive statistical analyses were performed using SPSS software.

**Results:**

Between 2022 and 2025, a total of 12,313 urological surgical procedures were performed at our center, among which 57 patients were newly diagnosed with postoperative LEVT during hospitalization, corresponding to an overall procedure-based clinically detected LEVT rate of 0.46% (57/12,313). The corresponding annual procedure-based clinically detected LEVT rates were 0.63% (17/2,711) in 2022, 0.43% (13/3,005) in 2023, 0.25% (8/3,256) in 2024, and 0.57% (19/3,341) in 2025, showing annual variation over the four-year study period. One patient was excluded due to archived medical records, and two patients were excluded due to incomplete data, leaving 54 patients for detailed analysis. Among these, 45 were male (83.33%) with a median age of 69.0 years (interquartile range, 61.0–74.75). Benign prostatic hyperplasia constituted the largest subgroup by primary condition (29/54, 53.70%), and transurethral resection of the prostate was the most common surgical procedure among the identified LEVT cases (28/54, 51.85%). Thrombosis predominantly involved the intermuscular veins (62.96%), followed by deep veins (35.19%). The median time to LEVT detection was 7 days after admission (IQR, 6–8) and 4 days after surgery (IQR, 3–5). Clinical manifestations were mainly local symptoms such as limb swelling or pain (79.63%). Anticoagulation with low-molecular-weight heparin was the primary treatment (70.37%), and most patients experienced no significant complications (92.59%).

**Conclusion:**

The overall procedure-based clinically detected postoperative LEVT rate during hospitalization in urological surgery was low, with annual variation observed over the four-year study period. Among the identified LEVT cases, benign prostatic hyperplasia was the most common primary condition, with intermuscular vein thrombosis being the most common type, and most cases were detected in the early postoperative period. Clinical attention should focus on continued perioperative VTE risk assessment and early recognition of clinically suspected LEVT.

## 1 Introduction

Venous thromboembolism (VTE) is a common and clinically significant perioperative complication, primarily comprising deep vein thrombosis (DVT) and pulmonary embolism (PE). A study including 253,212 patients undergoing major complex surgeries reported a perioperative VTE incidence of approximately 1.9%, with occurrence significantly associated with long-term risk of thrombus recurrence (1). Furthermore, another study encompassing over 12 million surgical patients demonstrated that perioperative PE, although relatively rare at 0.18%, substantially increases hospital length of stay, medical costs, and short-term mortality, highlighting its clear clinical impact (2).

Within the clinical spectrum of VTE, lower extremity venous thrombosis (LEVT) represents the most frequent manifestation. Importantly, LEVT is not confined to the deep venous system; it can also involve intermuscular and superficial veins, reflecting a continuous pathological process across anatomical layers (3). Previous studies have reported that, among hospitalized patients, distal DVT and mixed-type DVT are prevalent, with distal DVT accounting for approximately 43.5% and mixed-type DVT approximately 39.2%, while intermuscular vein thrombosis also shows a considerable proportion (∼28.7%) (4). These findings suggest that lower extremity venous thrombosis exhibits hierarchical anatomical distribution and morphological coexistence. Additionally, research on hospital-acquired lower extremity DVT has shown that intermuscular veins are involved in up to 87.5% of cases, far exceeding the involvement of proximal veins (7.3%), with distal DVT occurring significantly more frequently than proximal DVT (5). These results indicate that intermuscular veins may play an important role in thrombus formation and propagation toward proximal veins, supporting the concept of a “multi-level anatomical involvement spectrum” in lower extremity venous thrombosis.

Urological surgical patients are generally considered a high-risk population for thrombus formation, with risk driven by multiple factors, including advanced age, a higher burden of comorbidities, a relatively high proportion of malignancy, and perioperative immobilization (6). A large-scale study based on the ACS-NSQIP database, including 207,861 patients undergoing urological cancer surgery, reported a postoperative venous thrombotic event incidence of 1.2%, reaching up to 3.16% in patients undergoing radical cystectomy. Multivariable analysis further identified advanced age, obesity, blood transfusion, and comorbid conditions as important risk factors for postoperative thrombotic events (7).

Although perioperative pharmacological anticoagulation and mechanical prophylaxis have been widely implemented (8), several limitations remain. First, most existing studies rely on symptom-driven evaluation or imaging-confirmed diagnosis, with limited systematic characterization of postoperative first-detected LEVT in real-world clinical practice, which may lead to underestimation of its true clinical occurrence. Second, previous studies have primarily focused on overall VTE risk or conventional DVT outcomes, whereas systematic evaluation of the full spectrum of postoperative LEVT in urological patients—including deep, intermuscular, and superficial venous involvement—remains limited. Against this background, the present single-center real-world study retrospectively analyzed patients undergoing urological surgery between 2022 and 2025, focusing on cases of LEVT first identified during postoperative hospitalization. The study systematically described annual variation and clinical characteristics, aiming to provide evidence to support perioperative thrombotic risk recognition and stratified prevention strategies in urological surgical patients.

## 2 Methods

### 2.1 Study design

This single-center, retrospective, real-world observational study was conducted based on the hospital electronic medical record system. We retrospectively collected and analyzed data on postoperative LEVT in urological inpatients from January 2022 to December 2025, including annual variation in procedure-based clinically detected LEVT rates. Detailed patient-level information was further extracted for all eligible cases. All included cases represented LEVT first identified during postoperative hospitalization.

### 2.2 Study population

Patients were eligible if they underwent urological surgery at our center between January 2022 and December 2025 and were diagnosed with LEVT for the first time during postoperative hospitalization. Routine preoperative lower-extremity ultrasonographic screening was not performed in all patients. Therefore, in this study, “postoperative LEVT” refers to LEVT first identified after surgery and does not necessarily indicate thrombosis newly formed after surgery.

**Inclusion criteria:** (1) Underwent urological surgery at our center between January 2022 and December 2025; (2) First detection of LEVT during postoperative hospitalization; (3) Complete and accessible electronic medical records, laboratory tests, and imaging data; (4) Full perioperative care and treatment records available.

**Exclusion criteria:** (1) Preoperative diagnosis of LEVT; (2) Incomplete case records preventing extraction and analysis of key variables.

### 2.3 Data source and extraction

All study data were obtained from the hospital electronic medical record system. All urological surgical cases were exported in a standardized manner. The diagnosis of LEVT was primarily based on lower-extremity color Doppler ultrasonography, supplemented by other imaging modalities when necessary for comprehensive assessment. Lower-extremity ultrasonography was not routinely performed as systematic screening for all patients either before or after urological surgery. Instead, it was performed based on clinical indications, including symptoms suggestive of LEVT, such as lower-extremity pain or swelling, as well as laboratory abnormalities or other clinical findings that raised suspicion of thrombosis based on the treating physician's overall clinical assessment. Patients without relevant clinical indications did not routinely undergo postoperative lower-extremity ultrasonography. This clinically indicated imaging approach remained generally consistent throughout the study period from 2022 to 2025.

During the study period, our institution implemented a standardized VTE risk assessment and risk-stratified thromboprophylaxis protocol. All hospitalized patients underwent VTE risk assessment using the 2010 Caprini Risk Assessment Model (RAM) within 24 h of admission, with reassessment at relevant clinical time points, such as after surgery, when the clinical condition changed, upon transfer between departments, and at discharge. The initial assessment was based on risk factors available at the time of admission, whereas perioperative risk factors that became available or changed subsequently were incorporated during reassessment at the relevant clinical time points. Patients were classified as low risk (Caprini score 0–2), moderate risk (3–4), or high risk (≥5). Preventive strategies included basic prophylaxis, mechanical prophylaxis, and pharmacological prophylaxis, with specific measures selected according to the risk category and the individual clinical condition. Basic prophylaxis included VTE-related patient education, ankle-pump exercises, and encouragement of early mobilization. Mechanical prophylaxis included elastic compression stockings and intermittent pneumatic compression, while pharmacological prophylaxis was administered when clinically appropriate. Mechanical and pharmacological measures were therefore not routinely applied uniformly to all patients but were individualized according to risk assessment and clinical considerations. The overall VTE risk assessment framework and principles of risk-stratified thromboprophylaxis remained generally consistent from 2022 to 2025.

Data extraction was independently performed by two trained researchers and cross-checked for consistency. Any discrepancies were resolved with the assistance of a third researcher to ensure the accuracy and reliability of the extracted data.

### 2.4 Study variables

For the 54 patients included in the detailed case-level analysis, perioperative and clinical information was extracted and categorized into four domains as follows:

(1) General characteristics: including age, sex, body mass index (BMI), educational level, and comorbidities (such as hypertension, diabetes, and coronary artery disease), as well as history of thrombosis, family history of thrombosis, prior use of anticoagulant or antiplatelet therapy, smoking status, and alcohol consumption;
(2) Admission-related scores and laboratory tests: including functional status at admission and the initial Caprini risk assessment score obtained within 24 h of admission; laboratory parameters at admission, including complete blood count (hemoglobin, white blood cell count, and platelet count), liver and renal function tests (alanine aminotransferase, aspartate aminotransferase, albumin, total protein, creatinine, and blood urea nitrogen), coagulation profiles (prothrombin time, activated partial thromboplastin time, and international normalized ratio), and metabolic indicators including blood glucose and lipid profiles (total cholesterol, triglycerides, low-density lipoprotein cholesterol, and high-density lipoprotein cholesterol);
(3) Surgical-related variables: including type of surgery, surgical approach, type of anesthesia, operative duration, intraoperative blood loss, and intraoperative blood transfusion;
(4) LEVT-related characteristics: including thrombus type and anatomical location, time of LEVT detection (postoperative day and hospital day), clinical manifestations (e.g., limb swelling, pain, dyspnea, or asymptomatic presentation), treatment strategies, and clinical outcomes. Clinical outcomes were assessed during the index hospitalization and up to hospital discharge. Although all patients diagnosed with LEVT were advised to attend the vascular surgery outpatient clinic after discharge for continued evaluation and management of thrombosis, post-discharge outcomes were not systematically collected or analyzed in the present study. For descriptive analysis, thrombus classification was mutually exclusive and based on the most severe venous level involved as documented in the ultrasound reports. Patients with deep venous involvement were classified in the deep venous involvement category regardless of concomitant superficial or intermuscular vein thrombosis; patients with superficial venous involvement but no deep venous involvement were classified in the superficial venous involvement category; and patients with intermuscular vein thrombosis alone were classified in the intermuscular vein thrombosis category. Each patient was counted only once. Although calf muscular vein thrombosis is generally considered within the spectrum of distal DVT, intermuscular vein thrombosis was retained as a separate descriptive category in this study to preserve the anatomical information documented in the original ultrasound reports.

### 2.5 Quality control

All study data were obtained from the hospital information system. Medical records of patients with postoperative LEVT following urological surgery were retrieved and exported through a standardized query process.

After data extraction, repeated case verification, logical consistency checks, and outlier screening were performed by the research team. Key variables, including LEVT diagnosis, surgical procedure, operative duration, and time of LEVT detection, were cross-validated using electronic medical records, imaging reports, and laboratory test results to ensure data accuracy and completeness.

For cases with missing or incomplete information, analyses were conducted based on the principle of complete-case inclusion, using all available valid data for statistical evaluation.

### 2.6 Statistical analysis

Statistical analyses were performed using SPSS version 26.0. Categorical variables were presented as frequencies and percentages. Continuous variables were first assessed for normality using the Shapiro–Wilk test. Normally distributed variables were expressed as mean ± standard deviation, whereas non-normally distributed variables were summarized as median (P25, P75). As a retrospective descriptive study, only descriptive statistical methods were applied to summarize patient characteristics, admission-related scores, laboratory findings, surgical information, and LEVT-related variables. No between-group comparisons or inferential statistical analyses were performed. The procedure-based clinically detected LEVT rate during postoperative hospitalization was reported overall and by year.

### 2.7 Ethical statement

This study was approved by the Ethics Committee of our hospital (Approval No. S20260390-01). As a retrospective study, all data were anonymized prior to analysis. The study was conducted in accordance with the principles of the Declaration of Helsinki.

## 3 Results

### 3.1 Patient inclusion and overall rate of clinically detected LEVT

From 2022 to 2025, a total of 57 patients were identified with newly diagnosed postoperative LEVT following urological surgery. Among them, 1 case was excluded due to medical record restriction and 2 cases due to missing data. Finally, 54 patients were included in the final analysis. All included cases were patients undergoing urological surgery, and LEVT was diagnosed during postoperative hospitalization based on clinical evaluation, primarily supported by Doppler ultrasonography, rather than systematic screening.

During the same period, a total of 12,313 urological surgical procedures were performed. The overall procedure-based clinically detected postoperative LEVT rate was 0.46% (57/12,313). The annual surgical volume increased from 2,711 procedures in 2022 to 3,341 procedures in 2025, while the procedure-based clinically detected postoperative LEVT rate showed annual variation over the four-year study period. The annual procedure-based clinically detected LEVT rates were 0.63% (17/2,711) in 2022, 0.43% (13/3,005) in 2023, 0.25% (8/3,256) in 2024, and 0.57% (19/3,341) in 2025.

### 3.2 Baseline characteristics of patients

A total of 54 patients who were first diagnosed with lower extremity venous thrombosis after urological surgery were included. Of these, 45 were male (83.33%) and 9 were female (16.67%). In terms of educational level, junior high school was the most common (23 cases, 42.59%), followed by primary school (13 cases, 24.08%) and senior high school (8 cases, 14.81%); 7 patients (12.97%) had a university degree or above, and 1 case (1.85%) had missing information.

Regarding demographic characteristics, the median age was 69.00 (61.00, 74.75) years, height was 1.65 (1.60, 1.71) m, weight was 65.50 (57.38, 73.25) kg, and BMI was 23.90 (22.06, 26.85) kg/m^2^.

In terms of primary diagnosis, 29 patients (53.70%) had benign prostatic hyperplasia, 6 (11.11%) had urinary calculi, 6 (11.11%) had bladder cancer, 5 (9.26%) had renal cancer, and 3 (5.56%) had prostate cancer. The remaining cases included adrenal cortical adenoma, renal cyst, ureteral stricture, ureteral tumor, and scrotal tumor, each accounting for one case (1.85%).

Regarding comorbidity burden, 30 patients (55.56%) had ≤5 comorbid conditions, 18 (33.33%) had 6–10 comorbidities, and 6 (11.11%) had ≥11 comorbidities. For comorbid diseases, 33.33% had hypertension, 9.26% had diabetes mellitus, and 9.26% had coronary heart disease.

No patients had a history of venous thrombosis or a family history of thrombosis (0%). Four patients (7.41%) had a history of anticoagulant or antiplatelet drug use.

In terms of lifestyle factors, 35 patients (64.81%) were never-smokers, 9 (16.67%) had quit smoking, 7 (12.96%) were current regular smokers, and 3 (5.56%) smoked intermittently. Regarding alcohol consumption, 34 patients (62.96%) never drank alcohol, 3 (5.56%) had stopped drinking, 4 (7.41%) were regular drinkers, and 13 (24.07%) drank intermittently. The detailed information is shown in Table 1.

**Table 1:** Baseline characteristics of patients (*N* = 54).

Variable	Category	Frequency	Percentage (%)
Gender	Female	9	16.67
	Male	45	83.33
Age (years)	69.00（61.00，74.75）		
Height (m)	1.65（1.60，1.71）		
Weight (kg)	65.50（57.38，73.25）		
Body mass index (BMI, kg/m^2^)	23.90（22.06，26.85）		
Educational level	Illiterate	2	3.7
	Primary school	13	24.08
	Junior high school	23	42.59
	Senior high school	8	14.81
	University or above	7	12.97
	Unknown	1	1.85
Primary diagnosis	Benign prostatic hyperplasia	29	53.7
	Prostate cancer	3	5.56
	Urinary calculi	6	11.11
	Adrenal cortical adenoma	1	1.85
	Renal cyst	1	1.85
	Renal cancer	5	9.26
	Bladder cancer	6	11.11
	Ureteral stricture	1	1.85
	Ureteral tumor	1	1.85
	Scrotal tumor	1	1.85
Number of diagnoses	≤5	30	55.56
	6–10	18	33.33
	≥11	6	11.11
Hypertension	No	36	66.67
	Yes	18	33.33
Diabetes mellitus	No	49	90.74
	Yes	5	9.26
Coronary heart disease	No	49	90.74
	Yes	5	9.26
History of thrombosis	No	54	100
Family history of thrombosis	No	54	100
History of anticoagulant/antiplatelet use	No	50	92.59
	Yes	4	7.41
Smoking status	Never smoker	35	64.81
	Former smoker	9	16.67
	Current regular smoker	7	12.96
	Occasional smoker	3	5.56
Alcohol consumption	Never drinker	34	62.96
	Former drinker	3	5.56
	Current regular drinker	4	7.41
	Occasional drinker	13	24.07

### 3.3 Admission-related scores and laboratory findings

At admission, the median Barthel Index was 100.00 (93.75, 100.00), indicating generally good self-care ability among patients. The median initial Caprini score assessed within 24 h of admission was 2.00 (2.00, 3.00).

Laboratory findings showed that the median hemoglobin level was 143.50 (125.00, 154.00) g/L, white blood cell count was 6.71 (5.39, 8.11) × 10⁹/L, and platelet count was 189.50 (149.00, 242.25) × 10⁹/L. For renal function, serum creatinine was 79.95 (69.58, 95.08) μmol/L and blood urea nitrogen was 5.80 (4.71, 6.67) mmol/L.

Regarding liver function, alanine aminotransferase (ALT) was 19.00 (14.00, 26.50) U/L, aspartate aminotransferase (AST) was 22.00 (18.00, 27.00) U/L, total protein was 71.36 (68.45, 74.42) g/L, and albumin was 44.25 (40.84, 48.01) g/L. For coagulation parameters, prothrombin time (PT) was 11.00 (10.70, 11.40) s, activated partial thromboplastin time (APTT) was 26.95 (26.20, 28.86) s, and international normalized ratio (INR) was 1.01 (0.96, 1.04).

Blood glucose level was 5.47 (4.91, 6.32) mmol/L. Lipid profile showed that total cholesterol was 4.94 (4.03, 5.57) mmol/L, triglycerides were 1.17 (0.90, 1.68) mmol/L, low-density lipoprotein cholesterol (LDL-C) was 2.84 (2.36, 3.70) mmol/L, and high-density lipoprotein cholesterol (HDL-C) was 1.29 (1.11, 1.59) mmol/L.

Overall, there was inter-individual variability across laboratory indicators, with relatively wide distribution ranges observed in some parameters. The detailed results are presented in Table 2.

**Table 2:** Admission scores and laboratory test results of patients (*N* = 54).

Variable	N	M（P25，P75）	Range
Barthel Index (BI)	54	100.00（93.75，100.00）	23–100
Initial Caprini score (within 24 h of admission)	53	2.00（2.00，3.00）	0–7
Hemoglobin (Hb, g/L)	54	143.50（125.00，154.00）	95–171
White blood cell count (WBC, ×10⁹/L)	53	6.71（5.39，8.11）	3.32–22.15
Platelet count (PLT, ×10⁹/L)	54	189.50（149.00，242.25）	83–420
Serum creatinine (Scr, μmol/L)	54	79.95（69.58，95.08）	48.6–179.5
Blood urea nitrogen (BUN, mmol/L)	54	5.80（4.71，6.67）	3.27–10.0
Alanine aminotransferase (ALT, U/L)	53	19.00（14.00，26.50）	7–141
Aspartate aminotransferase (AST, U/L)	53	22.00（18.00，27.00）	12–50
Total protein (TP, g/L)	54	71.36（68.45，74.42）	57.43–87.46
Albumin (ALB, g/L)	54	44.25（40.84，48.01）	35.47–50.68
Prothrombin time (PT, s)	54	11.00（10.70，11.40）	9.2–14
Activated partial thromboplastin time (APTT, s)	54	27.00（26.20，28.86）	24.7–33.6
International normalized ratio (INR)	54	1.01（0.96，1.04）	0.85–1.29
Glucose (GLU, mmol/L)	53	5.47（4.91，6.32）	4.08–10.59
Total cholesterol (TC, mmol/L)	44	4.94（4.03，5.57）	1.84–7.66
Triglycerides (TG, mmol/L)	44	1.17（0.90，1.68）	0.53–4.17
Low-density lipoprotein cholesterol (LDL-C, mmol/L)	44	2.84（2.36，3.70）	0.71–5.30
High-density lipoprotein cholesterol (HDL-C, mmol/L)	44	1.29（1.11，1.59）	0.88–2.19

### 3.4 Surgical characteristics

Among the 54 patients with clinically detected LEVT, transurethral resection of the prostate (TURP) was the most common surgical procedure, performed in 28 cases (51.85%), followed by transurethral resection of bladder tumor in 7 cases (12.96%), lithotripsy for urinary calculi in 5 cases (9.26%), radical nephrectomy in 4 cases (7.41%), and radical prostatectomy in 3 cases (5.56%).

In terms of surgical approach, transurethral procedures accounted for the highest proportion (38 cases, 70.37%), followed by laparoscopic surgery (13 cases, 24.07%) and open surgery (3 cases, 5.56%).

Regarding anesthesia methods, general anesthesia was predominant (48 cases, 88.89%), while regional anesthesia and combined anesthesia were used in 2 cases (3.70%) and 4 cases (7.41%), respectively.

Perioperative transfusion was uncommon, with 53 patients (98.15%) receiving no intraoperative blood transfusion, and only 1 patient (1.85%) requiring transfusion.

Intraoperative indicators showed that the median operative time was 1.25 h (1.00, 2.04), and the median estimated blood loss was 50 mL (15, 80). Detailed information is presented in Table 3.

**Table 3:** Surgical characteristics of patients (*N* = 54).

Variable	Category	Frequency	Percentage (%)
Primary surgical procedure	Transurethral resection of the prostate (TURP)	28	51.85
	Transurethral resection of bladder tumor (TURBT)	7	12.96
	Ureteroscopic lithotripsy	5	9.26
	Radical nephrectomy	4	7.41
	Radical prostatectomy	3	5.56
	Ureteral stent placement	2	3.7
	Ureterovesical reimplantation	2	3.7
	Laparoscopic renal cyst decortication	1	1.85
	Adrenalectomy	1	1.85
	Scrotal cyst excision	1	1.85
Surgical approach	Open surgery	3	5.56
	Transurethral surgery	38	70.37
	Laparoscopic surgery	13	24.07
Anesthesia type	General anesthesia	48	88.89
	Regional anesthesia	2	3.7
	Combined anesthesia	4	7.41
Intraoperative blood transfusion	No	53	98.15
	Yes	1	1.85
Operation time (h)	1.25（1.00，2.04）		
Intraoperative blood loss (mL)	50（15，80）		

### 3.5 Thrombosis characteristics and treatment outcomes

Among the 54 patients who were first diagnosed with lower extremity venous thrombosis during hospitalization after surgery (confirmed by imaging examinations rather than systematic screening), only 2 patients (3.70%) had used elastic compression stockings for thromboprophylaxis before LEVT diagnosis, whereas 52 patients (96.30%) had not.

Thrombosis occurred predominantly in unilateral lower limbs (75.93%), while bilateral involvement accounted for 24.07%.

Based on the mutually exclusive classification used in this study, 34 patients (62.96%) had intermuscular vein thrombosis only, 1 patient (1.85%) had superficial venous involvement without deep venous involvement, and 19 patients (35.19%) had deep venous involvement.

Clinical manifestations were mainly local symptoms, with limb swelling or pain documented in 79.63% of patients, while systemic symptoms were documented in a small proportion (3.70%). For 9 patients (16.67%), typical LEVT-related symptoms were not clearly documented in the available medical records.

The median time to LEVT detection was 7 days after admission (6, 8) and 4 days after surgery (3, 5).

In terms of treatment, low-molecular-weight heparin was the most commonly used anticoagulation strategy (70.37%), followed by combined anticoagulation therapy (9.26%) and oral anticoagulants (5.56%). A small proportion of patients received interventional therapy (1.85%). Additionally, 7 patients (12.96%) did not receive specific anticoagulation therapy during hospitalization. In these patients, anticoagulation was temporarily withheld because of a high risk of postoperative bleeding and based on recommendations from vascular surgery consultation, and conservative management was adopted during hospitalization. All patients diagnosed with LEVT were advised to attend the vascular surgery outpatient clinic after discharge for continued evaluation and management of thrombosis.

During the index hospitalization and up to hospital discharge, most patients experienced no obvious complications (92.59%), while a small number developed adverse events such as bleeding, pulmonary embolism, or skin allergy. Post-discharge outcomes were not systematically collected or analyzed in this study. Detailed information is presented in Table 4.

**Table 4:** Thrombosis-related characteristics of patients (*N* = 54).

Variable	Category	Frequency	Percentage (%)
Thrombus location	Bilateral	13	24.07
	Unilateral	41	75.93
Type of thrombosis	Intermuscular vein thrombosis only	34	62.96
	Superficial venous involvement without deep venous involvement	1	1.85
	Deep venous involvement	19	35.19
Time from admission to LEVT detection (days)	7（6，8）		
Time from surgery to LEVT detection (days)	4（3，5）		
Thrombosis-related clinical symptoms	Typical LEVT-related symptoms not clearly documented	9	16.67
	Local symptoms (swelling/pain)	43	79.63
	Systemic symptoms (dyspnea/syncope, etc.)	2	3.7
Treatment of thrombosis	No specific anticoagulation (observation and follow-up)	7	12.96
	Oral anticoagulants	3	5.56
	Low-molecular-weight heparin	38	70.37
	Combination therapy (anticoagulation + oral agents)	5	9.26
	Interventional therapy	1	1.85
Use of elastic compression stockings for thromboprophylaxis before LEVT diagnosis	No	52	96.3
	Yes	2	3.7
In-hospital complications	None	50	92.59
	Bleeding	2	3.7
	Skin allergy	1	1.85
	Pulmonary embolism	1	1.85

## 4 Discussion

This study, based on single-center real-world data, conducted a descriptive analysis of postoperative in-hospital first-diagnosed LEVT in patients undergoing urological surgery. The results showed that the overall procedure-based clinically detected LEVT rate in our department was 0.46%, which was within the relatively low range reported in previous studies. However, the procedure-based clinically detected LEVT rate showed annual variation over the four-year study period. Given the limited number of annual observations and LEVT events, these variations should be interpreted cautiously and may reflect random variation as well as differences in surgical volume, patient composition, clinical imaging practices, unmeasured variation in the implementation of thromboprophylaxis, or clinical awareness. Although the general clinically indicated imaging approach remained consistent from 2022 to 2025, the overall and annual proportions of surgical patients undergoing lower-extremity ultrasonography could not be reliably determined; therefore, the potential influence of imaging practices on the observed annual detection rates cannot be completely excluded. A large-scale study including 902,968 surgical patients reported a postoperative VTE incidence of 0.83% (9). In addition, a retrospective study of Asian patients undergoing surgery for localized prostate cancer showed a relatively low incidence of perioperative VTE, with a 3-month incidence of 0.16% and a 2-year cumulative incidence of 0.29%–0.81% (10).

It should be noted that lower-extremity ultrasonography was not routinely performed as systematic screening for all patients either before or after surgery in the present study; rather, imaging was performed based on clinical indications, including symptoms suggestive of LEVT or other clinical findings that raised suspicion of thrombosis. Consequently, the reported procedure-based clinically detected LEVT rate primarily represents LEVT detected during postoperative hospitalization rather than the true overall incidence that might be observed with systematic ultrasonographic screening. Wang et al. reported that DVT was detected by ultrasonography within 7 days after surgery in 22 of 262 elderly women (8.4%) undergoing colpocleisis (11). In another study, routine preoperative lower-extremity compression ultrasonography identified LEVT in 117 of 1,253 women (9.3%) scheduled for pelvic organ prolapse surgery (12). Although these studies involved urogynecological rather than urological surgical populations, they illustrate that systematic ultrasonographic assessment may identify clinically silent thrombotic events, including occult preoperative LEVT. In the present study, patients with a documented preoperative diagnosis of LEVT were excluded; however, because routine preoperative ultrasonographic screening was not performed in all patients, the possibility that some thromboses first identified after surgery had already been present preoperatively cannot be excluded. Accordingly, the term “postoperative LEVT” in this study refers to LEVT first identified after surgery rather than thrombosis confirmed to have newly developed postoperatively.

This study showed that among 54 patients with postoperative first-diagnosed LEVT after urological surgery, based on the mutually exclusive classification used in this study, 34 patients (62.96%) had intermuscular vein thrombosis only, 1 patient (1.85%) had superficial venous involvement without deep venous involvement, and 19 patients (35.19%) had deep venous involvement. Although calf muscular vein thrombosis is generally considered within the spectrum of distal DVT, intermuscular vein thrombosis was retained as a separate descriptive category in the present study to preserve the anatomical information documented in the original ultrasound reports. The relatively large proportion of patients with intermuscular vein thrombosis alone highlights the frequent involvement of the calf muscular venous system among the clinically detected LEVT cases in this study, which is consistent with previous reports indicating that LEVT commonly involves calf veins (13). Anatomically and hemodynamically, the calf venous system, particularly the intermuscular veins, is highly dependent on the muscle pump for venous return and may therefore be susceptible to low-flow conditions and local blood stasis (3). Anatomical studies further suggest that the intermuscular venous system has a complex network of communicating branches and relies on coordinated muscle contraction and local fascial structures for venous return, with restriction of these structures potentially contributing to local blood stasis and thrombus formation (14).

In addition, a study of hospitalized patients with isolated calf deep vein thrombosis found that intermuscular veins, particularly the gastrocnemius and soleal veins, were the most frequently involved sites (52.6%), with thrombi also involving axial and intermuscular veins across different anatomical layers (15). In terms of clinical manifestations, LEVT in the present study was mainly detected in the early postoperative period, and most patients had documented limb swelling or pain. For some patients, typical LEVT-related symptoms were not clearly documented in the available medical records; therefore, these cases should not be interpreted as confirmed asymptomatic LEVT. Considering the uncertainty of its potential progression, continuous monitoring of dynamic changes in intermuscular thrombosis in patients after urological surgery may have potential clinical value.

This study showed that patients with postoperative LEVT after urological surgery were predominantly elderly males, with a median age of 69 years, reflecting the demographic distribution of the LEVT cases included in this study. In terms of primary disease composition, prostate hyperplasia–related surgery was the main category, and transurethral resection of the prostate accounted for more than 50% of the identified LEVT cases. However, because procedure-specific denominators for the overall urological surgical population were unavailable, the relatively large proportion of TURP among the identified LEVT cases should not be interpreted as indicating a higher procedure-specific LEVT risk. Furthermore, without a comparator group of patients without LEVT, it could not be determined whether older age, male sex, benign prostatic hyperplasia, or transurethral prostate surgery was associated with LEVT occurrence or merely reflected characteristics of the underlying urological surgical population. Previous studies focusing on transurethral resection of the prostate reported that among 464 patients, 5.4% developed thromboembolic events, of which 1.9% were DVT, and no pulmonary embolism was observed. Age, Caprini score, and operative time were reported to be associated with thrombosis in that study (16), but these associations could not be evaluated in the present descriptive study.

From the perspective of perioperative management, most patients in this study received general anesthesia and commonly experienced reduced postoperative mobility. Previous studies have suggested that anesthesia-related endothelial dysfunction, microcirculatory disturbance, and venous blood stasis may collectively contribute to the pathophysiological process of thrombosis (17, 18). Mechanical prophylactic measures, such as elastic stockings and intermittent pneumatic compression devices, are commonly recommended for perioperative VTE prevention. At our institution, VTE prevention was based on standardized risk assessment and risk-stratified selection of basic, mechanical, and pharmacological measures. However, the retrospective dataset did not systematically capture the actual use of individual prophylactic measures among all surgical patients; therefore, their implementation and potential influence on the observed LEVT detection rates could not be evaluated.

Regarding risk assessment, the median initial Caprini score assessed within 24 h of admission among patients with LEVT in this study was relatively low. However, because the study included only patients with clinically detected LEVT and no comparison group without thrombosis, this finding should be interpreted descriptively and does not permit conclusions regarding the predictive performance of the Caprini model or associations between individual risk categories and LEVT occurrence. The reported Caprini score represents the initial assessment within 24 h of admission rather than a postoperative or maximum score during hospitalization. It incorporated risk factors available at admission, while perioperative risk factors that emerged or changed subsequently, including those related to surgery, were incorporated during dynamic reassessment at relevant clinical time points. Thus, the relatively low initial score should not be interpreted as representing overall perioperative thrombotic risk. In clinical practice, continued dynamic risk assessment, together with individualized selection of basic, mechanical, and pharmacological prophylactic measures according to the patient's evolving clinical condition, remains important for perioperative VTE prevention.

## 5 Limitations

This study has certain clinical value; however, several limitations should be acknowledged. First, this was a single-center retrospective study with a relatively limited sample size, and the generalizability of the findings requires further validation. Second, not all patients underwent systematic preoperative and postoperative ultrasound screening; therefore, the possibility of pre-existing asymptomatic thrombosis cannot be completely excluded. Accordingly, “postoperative LEVT” in this study refers to LEVT first identified after surgery and does not necessarily indicate thrombosis newly formed after surgery. Because postoperative lower-extremity ultrasonography was clinically indicated rather than systematically performed, and the overall and annual proportions of surgical patients undergoing ultrasonography could not be reliably determined, the reported rates represent procedure-based rates of clinically detected LEVT rather than the true overall incidence. Although the general imaging approach remained consistent during the study period, variations in imaging practices may have influenced the observed annual detection rates. In addition, incomplete symptom documentation in some retrospective records precluded confirmation of the absence of typical LEVT-related symptoms, and the specific clinical or laboratory findings prompting ultrasonography could not be reliably reconstructed for all patients. Third, although a standardized VTE risk assessment and risk-stratified thromboprophylaxis protocol was implemented at our institution, the retrospective data did not systematically capture the actual use of individual mechanical and pharmacological prophylactic measures among all surgical patients; therefore, their overall and annual utilization and potential influence on LEVT detection rates could not be evaluated. Fourth, this study mainly adopted descriptive statistical methods and did not include a comparator group of patients without LEVT. Procedure-specific denominators for the overall urological surgical population were also unavailable, precluding calculation and comparison of procedure-specific LEVT detection rates. Therefore, the observed demographic, clinical, laboratory, and perioperative characteristics could not be interpreted as factors associated with LEVT occurrence. No risk factor analysis or predictive modeling was performed, and the findings should be interpreted descriptively rather than as evidence of associations or causal relationships. Fifth, clinical outcomes were assessed only during the index hospitalization and up to hospital discharge, and post-discharge outcomes were not systematically collected or analyzed. Therefore, subsequent thrombus progression, recurrence, pulmonary embolism, bleeding complications, and other thromboembolic outcomes could not be evaluated. Future multicenter, large-sample prospective studies using standardized screening strategies and appropriate comparator groups are needed to further explore the true incidence patterns and factors associated with LEVT after urological surgery.

## 6 Conclusion

This study showed that the overall procedure-based clinically detected LEVT rate during postoperative hospitalization among patients undergoing urological surgery was relatively low, with annual variation observed over the four-year study period. Among the mutually exclusive thrombus categories, intermuscular vein thrombosis alone represented the largest category, and most cases were detected in the early postoperative period, with clinical manifestations primarily presenting as local symptoms. In the perioperative management of patients undergoing urological surgery, attention should be paid to the early identification and dynamic monitoring of calf venous thrombosis, including intermuscular vein thrombosis, in addition to conventional deep vein thrombosis and pulmonary embolism. Future multicenter, large-sample prospective studies with standardized screening and appropriate comparator groups are needed to further clarify the detection patterns and associated factors of LEVT after urological surgery and inform the optimization of perioperative thromboprophylaxis strategies.

## Data Availability

The raw data supporting the conclusions of this article will be made available by the authors, without undue reservation.
